# Could Low Serum Albumin Level Be an Independent Marker of Severe Preeclampsia?

**DOI:** 10.3390/healthcare13131503

**Published:** 2025-06-24

**Authors:** Elena Ciciu, Andreea Alexandru, Bogdan Cimpineanu, Seila Musledin, Cristina Cioti, Camelia Pana, Alina Mihaela Stăniguț, Liliana-Ana Tuta

**Affiliations:** 1 Nephrology Department, Constanta County Emergency Hospital, 900591 Constanţa, Romania; elena.ciciu@365.univ-ovidius.ro (E.C.); alexandra_med16@yahoo.com (A.A.); bogdan.cimpineanu@365.univ-ovidius.ro (B.C.); tuta.liliana@365.univ-ovidius.ro (L.-A.T.); 2 Department of Clinical Medical Sciences, Faculty of General Medicine, “Ovidius” University of Constanţa, 900527 Constanţa, Romania; sheyla.ibadula@hotmail.com (S.M.); cioti.cristina.md@gmail.com (C.C.); 3 Doctoral School, Faculty of Medicine, “Ovidius” University of Constanţa, 900527 Constanţa, Romania

**Keywords:** preeclampsia, serum albumin, proteinuria, severity criteria, maternal complications, fetal outcomes

## Abstract

**Background**: Preeclampsia (PE) remains a significant cause of maternal and fetal morbidity and mortality, with diagnostic criteria still evolving. Serum albumin, a potential marker of endothelial dysfunction and protein loss, has been proposed as a severity indicator in PE. This study evaluates the clinical utility of serum albumin levels, particularly values below 2 g/dL, in assessing PE severity and predicting maternal–fetal complications. **Methods**: We conducted a prospective and descriptive study including 59 pregnant women diagnosed with PE. The participants were divided into mild (n = 23) and severe (n = 36) PE groups based on national guidelines. Serum albumin, 24 h proteinuria, renal and hepatic function markers, and fetal outcomes were analyzed. ROC curve analysis was employed to determine albumin’s diagnostic performance. **Results**: Serum albumin levels were significantly lower in the severe PE group compared to the mild PE group (1.82 ± 0.50 vs. 2.44 ± 0.36 g/dL, *p* < 0.001). ROC analysis identified a threshold of 2.3 g/dL (sensitivity: 88.9%, specificity: 73.9%) for distinguishing PE severity. A strong association was observed between albumin < 2 g/dL and severe proteinuria (>3 g/24 h), but no significant association emerged with renal or hepatic dysfunction, fetal complications, or birth outcomes. **Conclusions**: Although serum albumin < 2 g/dL is associated with severe proteinuria, it does not independently correlate with other maternal or fetal complications in PE. These findings suggest that albumin may serve as a complementary, but not a standalone, marker in assessing PE severity.

## 1. Introduction

Preeclampsia (PE) affects 5–8% of pregnancies worldwide and remains a major cause of both maternal and fetal morbidity and mortality [1,2,3]. According to the literature data, it is responsible for 40% of all preterm births [4]. Pregnant women with PE are prone to maternal or fetal complications (i.e., renal impairment, preterm birth, intrauterine growth restriction). Several studies have suggested that serum albumin and proteinuria are related to the severity of PE and its complications [5,6,7]. The Canadian Society of Obstetrics and Gynecology (SOGC) includes a serum albumin level below 2 g/dL as a possible severity criterion in preeclampsia, although current evidence remains limited, and this topic continues to be an area of active research [8].

Pregnancy with PE has a high risk of complications, and for this reason, researchers have analyzed different markers that could be associated with the severity of preeclampsia. The aim of this study was to establish a possible link between the severity of this pathology, the level of serum albumin, and the predictive value for the evolution of pregnancy and therapeutic results to reduce maternal or fetal complications. The results of this study could be useful for clinical practice, complementing existing studies and guiding clinicians towards higher accuracy of diagnosis regarding the certainty, severity criteria, and complications of PE.

## 2. Materials and Methods

Our study used a prospective and descriptive plan, in which 59 pregnant women with PE were included, between May 2019 and May 2024. All patients were admitted to Constanta County Emergency Clinical Hospital in the Gynecology Department and monitored by specialists from the Nephrology Department, with periodic follow-ups in the outpatient service of the hospital.

The included clinical variables were maternal age, gestational age at onset, severity, gestational age at birth, fetal birth weight and blood pressure values, maternal complications (eclampsia, HELPP syndrome), and fetal complications (prematurity, low birth weight). To complete the biological profile, we determined the following variables: proteinuria, serum albumin, uric acid, serum creatinine, platelet count, and liver enzymes. Their values were extracted from the electronic monitoring system of Constanta County Emergency Clinical Hospital. According to international guidelines, preeclampsia is classified as early-onset when diagnosed before 34 weeks of gestation and late-onset when it occurs at or after 34 weeks [8].

Severe PE met the following criteria [9,10,11]:

- Severe and persistent hypertension: SBP > 160 mmHg and/or DBP > 110 mmHg in 2 different situations and 4 h apart.
- Renal impairment, defined by doubling the serum creatinine level in the absence of other pathologies or a serum creatinine level > 1.1 mg/dL.
- Thrombocytopenia < 100.000/mm^3^.
- Hepatic cytolysis (transaminases > 70 U/L).
- Neurological complications: persistent headaches, visual disturbances, and progression to eclampsia (seizures).
- Utero-placental dysfunction: fetal growth restriction, oligo-hydramnios, abruptio placentae, or fetal death.

### 2.1. Inclusion and Exclusion Criteria of the Study Group

This study included pregnant women diagnosed with preeclampsia, with no history of essential hypertension, diabetes, or nephropathy, and aged over 16. Women were excluded if they had a gestational age below 20 weeks; chronic conditions, such as liver cirrhosis, heart failure, kidney transplant, or chronic kidney disease; and pre-existing essential hypertension, nephropathy, diabetes, insufficient follow-up, or incomplete biological data required for the study database.

### 2.2. Statistical Analysis

Experimental data were processed using IBM SPSS Statistics 23 and MedCalc 14.8.1. The procedures included descriptive statistics and graphs (Pie, bar+error bar). Parametric statistical tests (*t*-test for comparing the mean value of two independent samples). Nonparametric statistical tests were used to address categorical variables (χ^2^ test of association and of the link between two categorical variables; χ^2^ test and z-test for comparing two proportions), non-parametric statistical tests for normal data or to address numerical variables when the condition of normality was not satisfied (The median test), and ROC analysis. The significance level was α = 0.05. In each applied test, the null hypothesis was rejected if the statistical value of the test (tstat) was in the critical region (RC) or the associated probability with the statistical value of the test (p) was lower than α. In addition to descriptive and bivariate analyses, a multivariate logistic regression model was constructed to evaluate the independent association between serum albumin levels < 2 g/dL and the severity of preeclampsia. The dependent variables included serum albumin category, maternal age, gestational age at onset, and parity. Odds ratios (ORs) with 95% confidence intervals (CIs) were calculated. Statistical significance was considered at *p* < 0.05. The severity of preeclampsia was defined based on composite clinical criteria, including blood pressure levels, proteinuria, and the presence of maternal or fetal complications, according to national and international guidelines. In addition to descriptive and bivariate analyses, odds ratios (ORs) were calculated using logistic regression models. For this purpose, PE severity and serum albumin thresholds were treated as binary variables. Covariates such a gestational age, parity, and maternal age were included in the model based on clinical relevance.

## 3. Results

### 3.1. Distribution According to PE Severity

**Figure 1:**
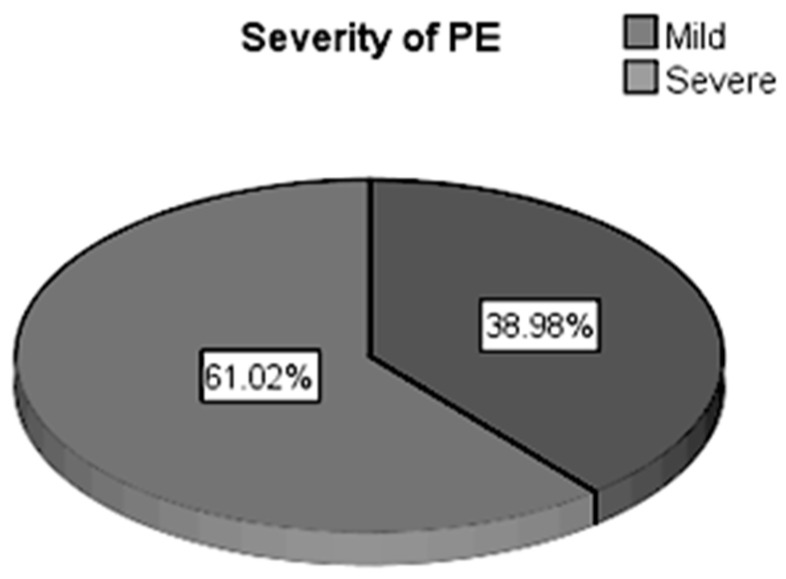
Distribution of pregnant women according to the degree of severity of PE.

Depending on the severity of PE, the study group included 23 (38.98%) pregnant women with mild PE and 36 (61.02%) pregnant women with severe PE, as shown in Figure 1. The 36 (61.02%) pregnant women with severe PE were classified according to the following severity criteria:

- Severe hypertension 47.2% (17 pregnant women).
- Severe proteinuria 52.8% (26 pregnant women).
- Alteration of liver function 38.9% (14 pregnant women).
- Alteration of renal function 30.6% (11 pregnant women).
- Severe thrombocytopenia 25% (9 pregnant women).

### 3.2. Distribution of Pregnant Women According to Gestational Age at Onset and Severity of Preeclampsia

The distribution of pregnant women according to the onset and severity of PE is summarized in Table 1. Depending on the gestational age at onset of PE, the study group was divided into two groups: early onset (<34 weeks) and late onset (>34 weeks). There is an association between the two variables mentioned in this study, i.e., the onset and degree of PE impairment: χ^2^_calc_ = 7.81, df = 1, and *p* = 0.0052.

The risk of finding severe PE in pregnant women with early onset is 5.625 times higher than the risk of finding severe PE in pregnant women with late-onset PE (OR = 5.625, 95% CI for OR = 1.794–17.633).

### 3.3. The Value of Serum Albumin Depending on the Severity of PE

**Figure 2:**
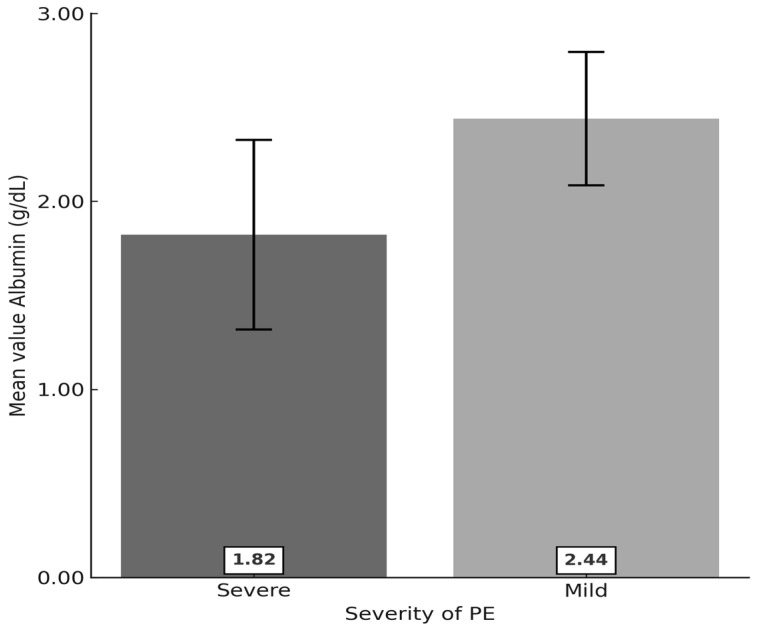
Bar and error bar graph of mean serum albumin values according to the severity of PE (error bars represent mean value ± SD).

Albumin levels were significantly different between the group with severe PE and the group with mild PE (2.44 ± 0.36 and 1.82 ± 0.50, respectively). The variations in the two groups were considered unequal (*p* < 0.001) (Table 2 and Figure 2).

### 3.4. The ROC Curve of Serum Albumin Used in Determining the Severity of Preeclampsia

**Figure 3:**
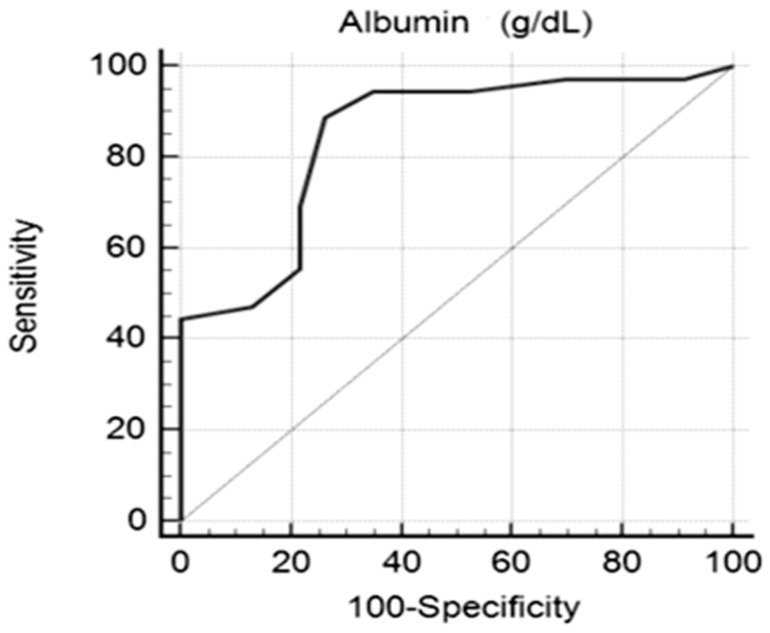
ROC curve of albumin in the study group with severe/mild PE.

To set up the performance of serum albumin in clinical practice, the ROC curve of serum albumin was created, distinguishing between the two groups with PE = severe/mild. The area under the ROC curve (AUC) was estimated to be 0.847 with a 95% confidence interval of (0.729–0.927). The statistical value of the test was z_statistic_ = 6.505, and the associated probability was *p* < 0.0001, which is why the area under the curve was different from 0.5. The Youden index was 0.6280, establishing a level of 2.3 g/dL, which represents the threshold between ***severe PE*** and ***mild PE*** (sensitivity 88.89%; specificity 73.9%), as shown in Figure 3. Although the ROC analysis identified an optimal cutoff of 2.3 g/dL for our population, the descriptive group comparisons used the 2 g/dL threshold to remain consistent with international clinical guidelines.

### 3.5. Association Between Serum Albumin and 24 h Proteinuria in PE

Depending on the serum albumin value, the group of pregnant women with PE was divided into the following:

- Mild hypoalbuminemia > 2 g/dL (34 pregnant women).
- Severe hypoalbuminemia < 2 g/dL (25 pregnant women).

The distribution of pregnant women according to the level of albumin and 24 h proteinuria in the group of pregnant women with PE is shown in Table 3. There is an association between the two variables used in this study: χ^2^_calc_ = 8.03, df = 1, and *p* = 0.0046.

According to the results in Table 3, the proportion of patients with **serum albumin > 2 g/dL** with proteinuria between 0.3 and 3 g/24 h, amounting 73.5%, 25 out of a total of 34, differs significantly from the proportion of patients with **serum albumin > 2 g/dL** with proteinuria higher than 3 g/24 h, amounting to 36.0%, 9 out of the total of 25 (*p* < 0.001). Similarly, the proportion of patients with **serum albumin < 2 g/dL** with proteinuria between 0.3 and 3 g/24 h, amounting to 26.5%, 9 out of the total of 34, differs significantly from the proportions of patients with **serum albumin < 2 g/dL** with proteinuria higher than 3 g/24 h, amounting to 64.0%, 16 out of the total of 25 (*p* < 0.001).

### 3.6. Clinical and Paraclinical Features of Severe PE According to Serum Albumin Values

To better understand which clinical elements of preeclampsia are associated with hypoalbuminemia, we performed secondary analyses exploring the relationship between serum albumin and individual features such as proteinuria, renal and hepatic dysfunctions, and fetal complications. The clinical and paraclinical characteristics of severe PE according to albumin values, subdivided into two groups values less than 2 g/dL and values higher than 2 g/dL are summarized in Table 4.

According to the data in Table 4, there are no significant differences in albumin values and gestational age at onset (*p* > 0.05). In addition, there is no association between albumin values and gestational age at onset with alteration of renal/liver function and proteinuria (*p* > 0.05).

The risk of encountering the impairment of renal function, liver function, and severe 24 h proteinuria in pregnant women with serum albumin > 2 g/dL is considered equal to the risk of encountering impairment of renal function, liver function, and severe proteinuria in patients with serum albumin < 2 g/dL. The risk associated with paraclinical data and serum albumin, divided into two subgroups, is summarized in Table 5.

#### Multivariate Analysis

In the multivariate logistic regression analysis adjusting for maternal age, gestational age at onset, and parity, serum albumin levels <2 g/dL were not found to be an independent predictor of severe preeclampsia (odds ratio = 1.26; 95% confidence interval: 0.46–3.44, *p* = 0.648). However, gestational age at onset remained significantly associated with severe PE (*p* = 0.030) (see Table 6).

### 3.7. Maternal and Fetal Complications in Preeclampsia and Correlation with Serum Albumin

According to data from Table 7, we observed the following:

- No significant differences in albumin levels depending on gestational age at birth and newborn weight (*p* > 0.05).
- No association between albumin levels and fetal complications, prematurity, and low birth weight of the newborn (*p* > 0.05).

The risk of fetal complications, prematurity, and low birth weight in pregnant women with serum albumin > 2 (g/dL) is considered equal to the risk of fetal complications, prematurity, and low birth weight in patients with serum albumin < 2 (g/dL). The risk of fetal complications associated with serum albumin levels, divided into two subgroups, are shown in Table 8.

## 4. Discussion

During pregnancy, serum albumin levels naturally decrease because of plasma volume expansion and increased metabolism and interstitial volume [12,13]. Moreover, levels are significantly lower in women with PE compared to healthy pregnant women [14,15]. In pregnant women with PE and proteinuria, there is a progressive decrease in serum albumin due to increased protein requirements, urinary protein losses, and the passage of albumin into the interstitial fluid due to endothelial damage. An increase in sensitivity to angiotensin II is present in these patients, which leads to vasoconstriction and increased blood pressure [5].

In normal pregnancy, urinary protein excretion increases, which is considered pathologic when it exceeds the limit of 300 mg/24 h [16]. In clinical practice, proteinuria is widely used in decision-making regarding the termination of pregnancy in pregnant women with PE and is included in the severity criteria of the SOGR 2019 national guidelines. A value above 3 g/24 h is considered a strong criterion for PE severity because it accelerates its progression, which is associated with multiple maternal and fetal complications [11,17]. These findings align with recent studies indicating that decreased serum albumin is associated with adverse fetal and maternal outcomes, including low birth weight and placental histopathological changes, suggesting its potential as a predictive biomarker in preeclampsia [18].

According to the statistical results in our study group, serum albumin values show significant differences between severe PE and mild PE (2.44 ± 0.36 and 1.82 ± 0.50, respectively). To determine the performance of serum albumin in clinical practice, the ROC curve of the serum albumin value was created, which established that a level of 2.3 g/dL represents the threshold between severe PE and mild PE (sensitivity 88.89%; specificity 73.9%). We observed a strong association between albumin < 2 g/dL and severe proteinuria (>3 g/24 h) (χ^2^_calc_ = 8.310, df = 1, *p* = 0.004 < α = 0.05). The proportion of pregnant women with serum albumin < 2 g/dL and proteinuria > 3 g/24 h (64% of 25 pregnant women) was significantly different from the proportion of pregnant women with serum albumin < 2 g/dL and proteinuria less than 3 g/24 h (26.5% of 34 pregnant women) (*p* < 0.001). The same result was also found in patients with nephrotic syndrome, where proteins are lost in the urine over several weeks [19], in contrast to PE, where this loss develops in a much shorter time. Our data were in accordance with the study by Lei et al., which showed that 24 h proteinuria and serum albumin were closely related to the severity of PE [20,21,22].

Of the 34 pregnant women with serum albumin > 2 g/dL, 16 presented at least one severity criterion from the national guidelines, and of the 25 pregnant women with serum albumin < 2 g/dL, 20 presented at least one criterion of severity of PE. We could not establish the value of serum albumin that can be associated with a decline in liver function or renal function. The association risk of serum albumin < 2 g/dL with PE severity criteria was equal to the association risk of serum albumin > 2 g/dL (renal function impairment OR = 1.061, 95% CI for OR = 0.255–4.412, alteration of liver function OR = 0.556, 95% CI for OR = 0.140–2.200).

Our data suggest that although serum albumin levels are lower in pregnant women with severe PE, the serum albumin threshold of 2 g/dL may not represent an independent severity criterion in severe PE, as it is associated with severe proteinuria but not with altered kidney or liver functions. This interpretation is supported by the fact that while albumin showed a statistically significant difference between mild and severe PE in global analysis, individual associations with specific clinical or paraclinical severity markers were inconsistent. This may reflect limited statistical power due to subgroup sizes or heterogeneity in clinical presentation and should be considered when interpreting these findings. Furthermore, although our ROC curve analysis identified an optimal cutoff of 2.3 g/dL for distinguishing severe from mild PE in this cohort, we used the clinically recognized 2 g/dL threshold in descriptive comparisons to maintain consistency with international guidelines. These observations are consistent with recent evidence suggesting that a significant reduction in serum albumin during pregnancy may act as an early warning signal for the development of preeclampsia and is correlated with an increased risk of maternal and fetal complications [23].

Therefore, we question the inclusion of a serum albumin level below 2 g/dL in the list of criteria for severity of PE, a conclusion which is consistent with the studies performed by Benoit et al. [5].

Although serum albumin < 2 g/dL was associated with severe proteinuria, and lower albumin levels were observed among women with severe preeclampsia, our multivariate analysis showed that hypoalbuminemia was not an independent predictor of PE severity after adjusting for maternal age, gestational age at onset, and parity. These findings suggest that serum albumin may reflect disease burden but should not be interpreted as an independent severity marker without considering other clinical variables. However, other research supports the idea that alterations in serum albumin, as part of oxidative stress responses, could contribute to the pathophysiology of preeclampsia and may hold diagnostic value when combined with other biomarkers [24]. In this regard, recent efforts have focused on multimarker models for improving prediction accuracy. For example, Chirila et al. developed a second-trimester algorithm that combines the sFlt−1/PIGF ratio with uterine artery Doppler indices, significantly enhancing risk stratification for hypertensive disorders of pregnancy, as demonstrated in both an original study [25] and a recent review [26].

The evidence available in the literature shows that there is an association between serum albumin and the severity of PE [5]. In accordance with these data, Seong et al. [6] showed higher maternal and fetal morbidity among pregnant women with albuminemia < 3 g/dL. On the other hand, the PIERS study evaluated the serum albumin threshold of 1.8 g/dL in the prediction of severe maternal complications; the researchers of that study did not observe an association (*p* = 0.328 versus 0.483) [27]. Our conclusion was that low serum albumin levels cannot be used either as a significant predictor of the severity of preeclampsia or for the occurrence of these complications [28].

Our findings reveal that the proportion of newborns with low birth weight (1482.6 ± 437 g) and prematurity (31.20 ± 2.42 weeks) in pregnant women with PE and albuminemia < 2 g/dL did not reach statistical significance compared to their proportion in pregnant women with PE and serum albumin > 2 g/dL, having a difference of 225 ± 56 g in newborn weight and 1.43 weeks in gestational age at birth (χ^2^_calc_ = 1.702, df = 1, *p* = 0.192 and χ^2^_calc_ = 0.655, df = 1, *p* = 0.418, respectively). Our data failed to show an association between the lower than 2 g/dL serum albumin threshold and the occurrence of fetal complications: the encountered risk was equal between the two subgroups (fetal complication OR = 0.368, 95% CI for OR = 0.030–4.478, prematurity OR = 0.228, 95% CI for OR = 0.021–2.441, and low birth weight OR = 0.368, 95% CI for OR = 0.030–4.478). Thus, it was not possible to establish an albumin threshold to which fetal complications, such as low birth weight and prematurity, are frequently associated, discordant with some of the existing data. For example, the studies by Gojinic et al. and Seong et al. [6,7] stated that a level below 3 g/dL of serum albumin could correlate with these complications, representing a strong indicator of severity. Our findings differ from the studies by Seong et al. and Gojnic et al., which reported a stronger association between hypoalbuminemia and organ dysfunction. Similarly, some recent research has described adverse neonatal outcomes, such as lower birthweight and other complications, although statistical significance may not always be achieved in smaller cohorts [29]. These discrepancies may be explained by differences in study design, sample size, or clinical criteria used to define severity.

The maternal complications identified in our study included eclampsia, observed in four patients (6.8%), and HELLP syndrome, which occurred in one patient (1.7%). Other severe maternal complications associated with PE are well documented in the literature, although they were not observed in our study. This underscores the need for appropriate management to reduce maternal and fetal risk.

### Strengths and Limitations

This study addresses an important clinical gap by investigating serum albumin as a potential complementary marker for preeclampsia severity, in a context where reliable biomarkers remain limited. The prospective design, strict eligibility criteria, and comprehensive statistical analyses strengthen the validity of the findings. The data presentation, including ROC curve analysis and subgroup evaluations, is clear and facilitates potential clinical application. Moreover, the discussion responsibly integrates the results within the broader body of existing research, avoiding overinterpretation.

Nevertheless, some limitations should be noted. The single-center design and relatively small sample size may affect the generalizability of the findings. Including patients from additional hospitals would enhance external validity and allow for broader subgroup analyses. Additionally, the low incidence of severe maternal complications limited the ability to establish broader associations. Moreover, the absence of additional potential cofounding variables, such as body mass index (BMI) or pre-existing comorbidities, limits the ability to fully assess the independent predictive value of serum albumin. Further multicenter studies with larger cohorts are needed to better clarify the predictive role of hypoalbuminemia within preeclampsia management strategies.

## 5. Conclusions

In our cohort, serum albumin levels were significantly lower among women with severe preeclampsia compared to those with mild disease, and a threshold of 2.3 g/dL demonstrated good sensitivity and specificity in discriminating PE severity. However, after multivariate adjustment for maternal age, gestational age at onset, and parity, a serum albumin level below 2 g/dL was not independently associated with severe PE. Instead, hypoalbuminemia remained primarily associated with severe proteinuria and not with other recognized severity markers, such as renal or hepatic dysfunctions, or adverse fetal outcomes.

Therefore, while hypoalbuminemia may reflect disease burden, it should be considered as a complementary marker, a variable depending on clinical circumstances, particularly when interpreted alongside other clinical features. Further studies incorporating larger cohorts and more comprehensive multivariable modeling are warranted to better define the prognostic value of hypoalbuminemia in preeclampsia.

## Figures and Tables

**Table 1:** Distribution of pregnant women according to the onset and degree of PE severity.

		Severity of PE		
PE Onset		Severe	Mild	Total
Early onset	No. of pregnancies	27	8	35
(<34 weeks)	% Depending on onset	77.1%	22.9%	100.0%
	% Depending on severity	75.0%	34.8%	59.3%
Late onset	No. of pregnancies	9	15	24
(>34 weeks)	% Depending on onset	37.5%	62.5%	100.0%
	% Depending on severity	25.0%	65.2%	40.7%
	No. of pregnancies	36	23	59
Total	% Depending on onset	61.0%	39.0%	100%
	% Depending on severity	100%	100%	100%

**Table 2:** Albumin level depending on the severity of PE.

Severity of PE	Serum Albumin (g/dL)
Mild PE	2.44 ± 0.36
Severe PE	1.82 ± 0.50
*t*-test for independent samples	*t* = −5.507, df = 56.381, *p* < 0.001
Levene test for equality of variance	F = 8.72, *p* < 0.001

**Table 3:** Association and distribution of pregnant women with PE according to serum albumin and 24 h proteinuria.

		24 h Proteinuria		
Serum Albumin		0.3–3 g/24 h	>3 g/24 h	Total
	No. of pregnancies	25	9	34
>2 g/dL	% Depending on onset	73.5%	26.5%	100%
	% Depending on severity	73.5%	36%	57.6%
	No. of pregnancies	9	16	25
<2 g/dL	% Depending on onset	36%	64%	100%
	% Depending on severity	26.5%	64%	42.4%
	No. of pregnancies	34	25	59
Total	% Depending on onset	57.6%	42.4%	100%
	% Depending on severity	100%	100%	100%

**Table 4:** Clinical biological characteristics of PE according to serum albumin levels.

		Serum Albumin (g/dL)		
		>2	<2	*p*
Nr. of pregnant women with PE/severe PE		34/16	25/20	
Gestation age at the onset of PE (weeks) *		32.63 ± 2.5	31.20 ± 2.42	t = 1.730, df = 34, *p* = 0.093 ^a^ F = 0.127, *p* = 0.723 ^b^
Onset < 34 weeks. (no. of pregnant women %)		10 (37%)	17 (63%)	χ^2^_calc_ = 2.400, df = 1, *p* = 0.121
Severity criteria	Decline in Renal function (no. patient %)	5 (45.5%)	6 (54.5%)	χ^2^_calc_ = 0.007, df = 1, *p* = 0.936
	Hepatocytolysis	5	9	χ^2^_calc_= 0.707, df = 1,
	(no. of patient %)	(35.7%)	(64.3%)	*p* = 0.400
	Proteinuria	9	17	χ^2^_calc_ = 3.662, df = 1,
	(no. of patient %)	(34.9%)	(65.4%)	*p* = 0.056

(^a^ *t*-test for independent samples; ^b^ Levene’s test for equality of variances; * mean value +/− standard deviation; *p* < 0.05 statistically significant).

**Table 5:** Association risk of albumin levels with other paraclinical findings.

	Association Risk of Albumin Level > 2 g/dL vs. <2 g/dL	
Decline in Renal function	equal	OR = 1.061, 95% CI pt. OR = 0.255–4.412
Abnormal Liver function	equal	OR = 0.556, 95% CI pt. OR = 0.140–2.200
Severe level of 24 h proteinuria (nephrotic range)	equal	OR = 0.227, 95% CI pt. OR = 0.047–0.062

**Table 6:** Logistic regression analysis assessing predictors of PE severity.

Variable	OR	95% CI	*p*-Value
Albumin < 2 g/dL	0.23	−0.77–1.24	0.648
Maternal Age	0.04	−0.02–0.11	0.172
Gestational Age at Onset	−0.14	−0.27–−0.01	0.03
Parity	−0.49	−1.31–0.34	0.248

**Table 7:** Maternal and fetal complications in severe PE depending on albumin levels.

	Serum Albumin (g/dL)		*p*
	>2	<2	
No. of pregnant women with fetal complications	14 (87.5%)	19 (95.0%)	χ^2^_calc_ = 0.655, df = 1, *p* = 0.418
Gestational age at birth (weeks) *	32.63 ± 2.5	31.20 ± 2.42	t = 1.843, df = 34, *p* = 0.074 ^a^ F = 0.011, *p* = 0.915 ^b^
Prematurity	13 (81.3%)	19 (95%)	χ^2^_calc_ = 1.702, df = 1, *p* = 0.192
Weight of the newborn at birth (g) *	1707.5 ± 493.89	1482.6 ± 437	t = 1.447, df = 34, *p* = 0.157 ^a^ F = 0.243, *p* = 0.625 ^b^
Low weight at birth (g)	14 (87.5%)	19 (95%)	χ^2^_calc_ = 0.655, df = 1, *p* = 0.418
No. of pregnant women with maternal complications	1 (6.3%)	3 (15%)	not applicable for statistical analysis
Eclampsia	1 (6.3%)	3 (15%)	not applicable for statistical analysis
HELLP syndrome	0	1 (5%)	not applicable for statistical analysis

(^a^ *t*-test for independent samples; ^b^ Levene test for equality of variance; * median value+/− standard deviation, *p* < 0.05 significant different).

**Table 8:** The risk of fetal complications associated with albumin levels.

	Associated Risk of Albumin Level > 2 g/dL vs. <2 g/dL	
Fetal complications	equal	OR = 0.368, 95% CI pt. OR = 0.030–4.478).
Prematurity	equal	OR = 0.228, 95% CI pt. OR = 0.021–2.441
Low birth weight	equal	OR = 0.368, 95% CI pt. OR = 0.030–4.478

## Data Availability

The data are available on reasonable request from the corresponding author.
